# Molecular and Sensory Basis of a Food Related Two-State Behavior in *C. elegans*

**DOI:** 10.1371/journal.pone.0007584

**Published:** 2009-10-23

**Authors:** Juliette Ben Arous, Sophie Laffont, Didier Chatenay

**Affiliations:** 1 Laboratoire de Physique Statistique de l′Ecole Normale Supérieure, Centre National de la Recherche Scientifique (CNRS), Université Pierre et Marie Curie, Paris, France; 2 Laboratoire Jean Perrin, Centre National de la Recherche Scientifique (CNRS), Université Pierre et Marie Curie, Paris, France; Mount Sinai School of Medicine, United States of America

## Abstract

Most animals display multiple behavioral states and control the time allocation to each of their activity phases depending on their environment. Here we develop a new quantitative method to analyze *Caenorhabditis elegans* behavioral states. We show that the dwelling and roaming two-state behavior of *C. elegans* is tightly controlled by the concentration of food in the environment of the animal. Sensory perception through the amphid neurons is necessary to extend roaming phases while internal metabolic perception of food nutritional value is needed to induce dwelling. Our analysis also shows that the proportion of time spent in each state is modulated by past nutritional experiences of the animal. This two-state behavior is regulated through serotonin as well as insulin and TGF-beta signaling pathways. We propose a model where food nutritional value is assessed through internal metabolic signaling. Biogenic amines signaling could allow the worm to adapt to fast changes in the environment when peptide transcriptional pathways may mediate slower adaptive changes.

## Introduction

Most animals in their natural environment usually divide their time into two major states, an active moving state and an inactive resting state. Interestingly, across a large range of species the time spent in the inactive phase is usually much larger than the time spent moving actively [1]. In the laboratory, mice also alternate between two activity states even when there is no food shortage. In their home cages, wild type mice spend 66% of their time in inactivity [2]. Moreover, this time allocation is biologically regulated and may be crucial for the animal survival. For example mice mutants of the adipocyte hormone leptin or the 5-HT2C serotonin receptor display altered time budgets and obesity problems [2]. This type of intermittent behavior seems tightly linked to foraging and food consumption. Animals may therefore have to adapt their time allocation according to the availability of food in their environment.

We chose the soil nematode *Caenorhabditis elegans* as a model organism to study the molecular and environmental basis of this behavior. Its short life span and small size make it a tractable model to measure quantitatively long time scale behaviors such as time allocation in a range of different controlled environments. *C. elegans* is also a good model to study molecular basis of such behaviors, as the genetics of many worm behaviors have been studied in great details [3]–[5]. Moreover, *C. elegans* alternates between inactive and active phases on its standard food, the *Escherichia coli* strain OP50. In standard conditions Fujiwara et al estimated that *C. elegans* spends 80% of its time in an inactive mode called dwelling and 20% in an active mode called roaming [6]. During dwelling worms keep a low speed, alternate frequently between backward and forward movement and do not show much overall displacement. On the contrary roaming consists of straight displacements of sustained sinusoidal forward movement with few changes of direction. Moreover *C. elegans* modulates its time budget depending of the nutritional quality of the food present in its environment. The time allocated to roaming is smaller when the growth of the animal is favored by the quality of the food available [7]. This may be a strategy to select the best food environments and to leave areas where the quality of food is not sufficient for a sustainable population growth.

Here we show that *C. elegans* time allocation changes in response to the concentration of available food. Ultimately, upon food shortage, worms allocate all their time to the active phase. We provide evidence that internal signaling of food presence is necessary to induce the dwelling state and that, in presence of food, external sensory perception and previous starvation experiences modulate the time allocated to each phase. Finally, we show that serotonin, TGF-beta and insulin signaling control the time allocation in *C. elegans*.

## Results

### Wild type *C. elegans* exhibits two activity phases on food

**Figure 1:**
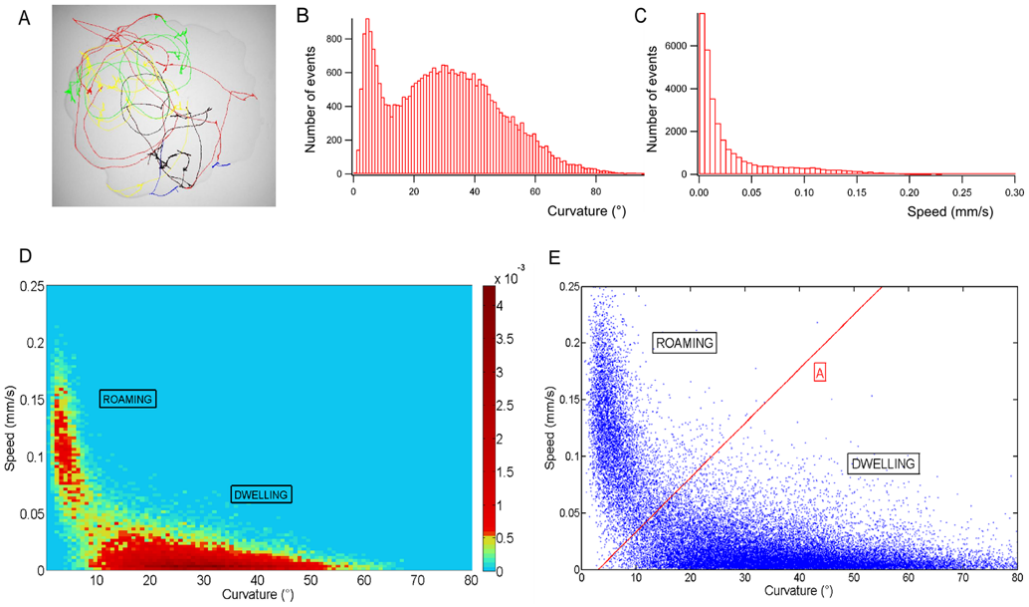
Two-state behavior of wild type *C. elegans* on food. (A) Trajectories of multiple WT *C. elegans* on OP50 (60 mm plate) (B) Curvature histogram for all the wild type worms (1bin = 1°) (C) Speed histogram for all the wild type worms (1bin = 0.05 mm/s) (D) Data points density map in the (Speed, curvature) plane. Two clusters can be observed (Data from 38 worms) (E) Clustering method. Data points over line A are attributed to the roaming phase, data points under line A to the dwelling phase. Line A has been determined on total WT data and is used for analysis of all the experiments in all the conditions.

To characterize *C. elegans* locomotory behavior on food, we used a custom automatic tracking system. We recorded multiple animals trajectories at 20°C for two hours with a 400 ms time resolution, on standard NGM plates evenly spread with the *E. coli* strain OP50 (Fig. 1A). The same method was used for all the following conditions and genotypes.

We were interested in long time scale locomotory behavior of the worm on food to determine whether we could quantify distinct activity phases. In order to filter out the sinusoidal and other short time scale features of the worm locomotion we split each worm trajectory in 10 seconds intervals and we calculated averaged speed and curvature for each of these short trajectory sections. The time period of the worm sinusoid on food lasts for about 4 seconds in our experiments. Thus, we used 10 s long sections as a trade off between keeping enough time resolution and erasing short-term features of the worm locomotion. We combined all the data points of the wild type worms, which allowed us to cluster quantitatively two behavioral states in the plane defined by speed and average curvature (Fig. 1).

Despite a quite different analysis method, we observed as in previous studies that wild type *C. elegans* display two different activity phases on food [6], [7]. We could observe a first cluster defined by trajectory sections of high speed and low average curvature. This cluster corresponds to the previously proposed definition of roaming as long slightly curvy movement with a few reorientations, and represents 20% of the events [6]. The roaming percentage for a given experiment was calculated as the number of points in the roaming cluster divided by the total number of points. We also observed another cluster defined by low speed and high average curvature events. This cluster corresponds to the inactive dwelling phase that is a succession of very short runs and many reversals. In our conditions N2 worms spent 80% of their time dwelling and did not show any quiescence events, which may be due to the quite low quality of the OP50 bacteria [8].

### *C. elegans* adapts its roaming behavior to food concentration

**Figure 2:**
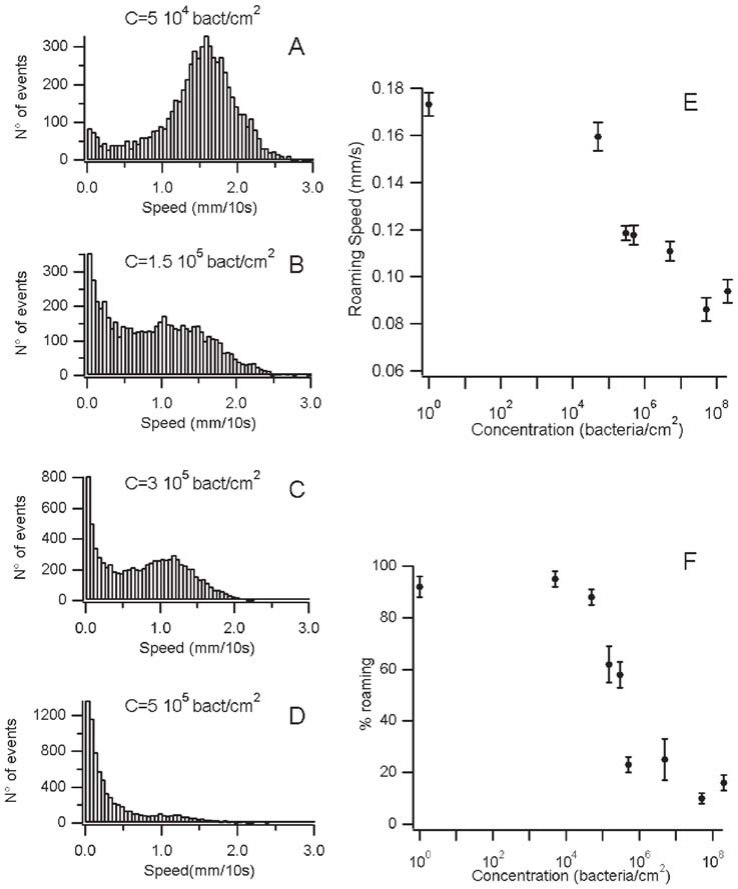
*C. elegans* adapts its roaming behavior to food concentration, both by changing the dwelling/roaming percentage and the speed during the roaming phase. (A–D) Speed histograms of WT *C. elegans* on different concentration of bacteria (A: 5 10^4^ B: 1.5 10^5^ C: 3 10^5^ D: 5 10^5^ bacteria per cm^2^, at least 15 worms have been recorded per condition, results are presented as mean ± s.e.m.) (E) Speed in the roaming phase decreases with food concentration (F) The percentage of time spent in the roaming phase is a function of food concentration (logarithm scale). Worms switch from an all-roaming to mostly dwelling in one decade of food concentration.

At high food concentration worms dwell most of their time. Besides, dwelling has not been observed off food and the proportion of time spent dwelling depends on food quality [7]. Thus, *C. elegans* seems to activate roaming in bad nutritional conditions. We wondered how the worm would behave at intermediate food concentrations and which concentration of bacteria was necessary to initiate the dwelling behavior. We analyzed trajectories of wild type worms without food and on a large range of food concentrations (from 0 to 10^8^ bacteria per cm^2^). Off food and at low bacteria concentration (up to 10^4^ bacteria per cm^2^) we found only one cluster corresponding to the roaming behavior. Above 5 10^5^ bacteria per cm^2^ worms behaved mostly as if they were in standard conditions and dwelled most of their time. Worm behavior switched from fully roaming to mostly dwelling over a range of bacterial concentrations (Fig. 2A–D, 2F). We could also detect a second effect of food concentration. Average speed in the roaming phase decreased when food concentration increased (Fig. 2E). Thus *C. elegans* roams less and at a lower speed as food concentration increases.

### Dwelling is not induced by external sensory perception

Worms can sense different food features, through chemosensory and mechanosensory circuits, but also through internal metabolic signaling reflecting their satiation state [9]–[13]. As a high food concentration is necessary to induce dwelling, we wanted to assess which components of food perception were needed, using a method developed by Gruninger & al [14]. They use bacteria treated with a low concentration of aztreonam, which is an antibiotic that prevents the septum closure during the division of *Escherichia Coli*. The last step of bacterial division is then blocked and *E. coli* that have been treated grow in long chains of undivided bacteria (up to 50 µm long). Gruninger & al have shown that the treated OP50 bacteria transmit mechanosensory and chemosensory clues comparable to regular OP50 signals but are of very low nutritional value to *C. elegans*. Worms can actually not swallow these bacteria because of their size [14].

**Figure 3:**
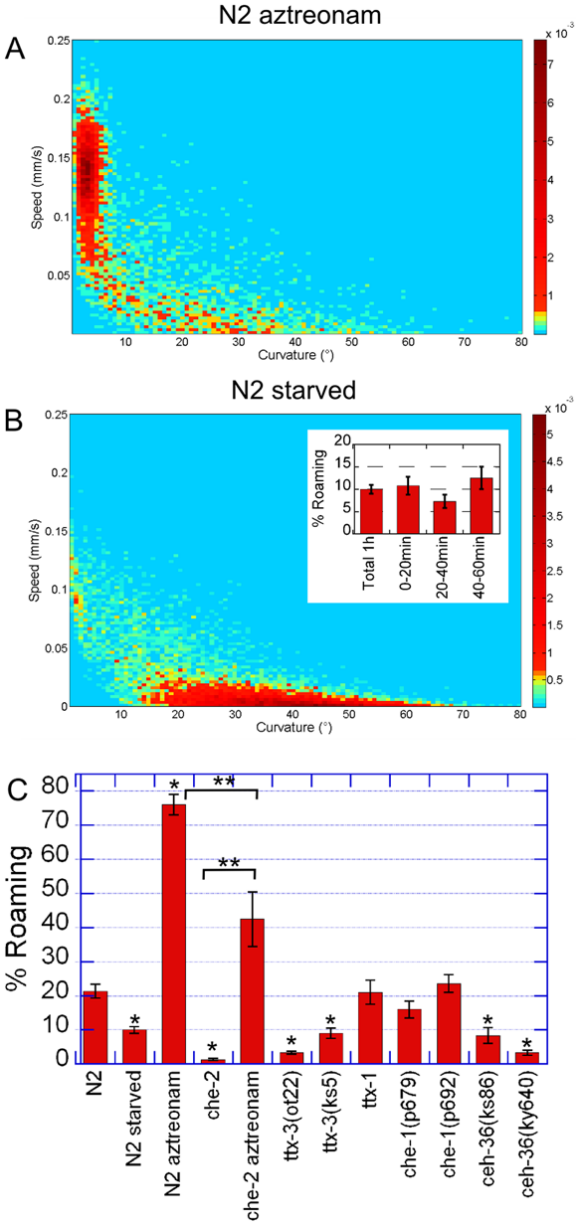
Influence of food perception and experience. (A–B) Data density maps for (A) N2 worms feeding on aztreonam treated bacteria. Compared to N2 worms in standard conditions (Fig 1-D) the dwelling cluster is suppressed (B) N2 worms feeding on standard OP50 bacteria after a 1 h fasting period. The roaming cluster is suppressed. Insert: Proportion of time spent roaming during the 0–20 min, 20–40 min and 40–60 min time intervals after a one-hour starvation period. (C) Quantitative measurements of the percentage of time spent roaming of N2 worms in aztreonam (A) and fasting (B) conditions and of mutants that have sensory perception defects. At least 20 worms were recorded per condition. Results are presented as mean ± s.e.m. *Different from wild type, p<0.01 **Different from each other p<0.01.

Wild type *C. elegans* spend 75% of their time roaming on aztreonam treated bacteria (Fig. 3A, 3C). On a standard bacterial lawn worms would show this phenotype on low concentrations of bacteria (∼10^5^ bacteria per cm^2^) (Fig. 2F). On aztreonam plates worms roam most of the time even though there is a high concentration of growing bacteria (∼10^7^ bacteria per cm^2^, similar to standard conditions). Thus, worms behave as if they could not perceive food presence correctly when they cannot swallow that food, even when they sense food chemosensory signaling. Thus, the signal needed for dwelling is probably a metabolic signal arising after digestion. External sensory perception of food is then not sufficient to stimulate dwelling and internal metabolic signaling is the major component of food perception necessary to induce this behavior.

This finding is also consistent with the behavior of cilium defective *che-2* mutants. These mutants have altered sensory perception and are mostly unable to roam on food. These mutants show normal locomotory behavior when stimulated and do not have basic locomotory defects [6]. On aztreonam treated bacteria, they roam much more than on regular food but still spend significantly less time in the roaming phase than wild type worms (Fig. 3C). These worms are then still able to respond to the presence of nutritional food but are defective in roaming in both conditions. Thus, external sensory perception seems to promote roaming in favorable and unfavorable food conditions.

Some specific amphid neurons mutants also show altered roaming behavior on food (Fig. 3C). AIY and ASI amphid neurons have been shown to extend roaming periods on food [7]. We checked mutants of neuronal terminal selector genes, which are transcription factors necessary for the development of specific sensory neurons [15]. We analyzed the behavior of worms with defects in the AWC olfactory neurons, the AFD thermosensory neurons or the gustative ASE neurons. All of these neurons have projections on the AIY interneurons. *ceh-36* (AWC+ASE) and *ttx-3*(AIY) mutants roamed twice less than wild type worms on food, even though they were able to move when stimulated. On the contrary *ttx-1*(AFD) and *che-1*(ASE) did not show any significant phenotype for this behavior (Fig. 3C). Therefore chemosensory perception through the AWC neurons seems to be necessary to maintain roaming phases on food. However, the behavior of wild type *C. elegans* on aztreonam treated bacteria shows that sensory perception is not sufficient to induce the transition from an all-roaming phenotype to a two-state behavior when worms encounter food.

### Previous starvation experience enhances dwelling behavior

After a starvation period, *C. elegan*s displays an “enhanced slowing response” when entering a bacterial lawn. This behavior is mediated by the neuromodulator serotonin [11]. The worm previous experience can then modulate its feeding behavior when it reencounters a food source.

In order to investigate how starvation affects the dwelling and roaming behavior, we starved wild type animals for an hour then transferred them to standard seeded plates. We recorded and analyzed one hour-long trajectories of these fasted animals on bacteria.

Starved worms roam twice less than well-fed worms. The proportion of time spent dwelling is mostly constant through the one-hour recording (Fig. 3B–C). This result shows that the enhanced slowing response can last for at least an hour after reencountering food.

### Serotonin mutants are defective in roaming behavior and do not respond to starvation

As biogenic amines signaling pathways have been previously implicated in food related behaviors of *C. elegans* [10], [11], [13], [16], it appeared interesting to test the importance of these signaling pathways on the roaming and dwelling behavior. Therefore, we analyzed the trajectories of different mutants defective in these pathways in standard conditions.

**Figure 4:**
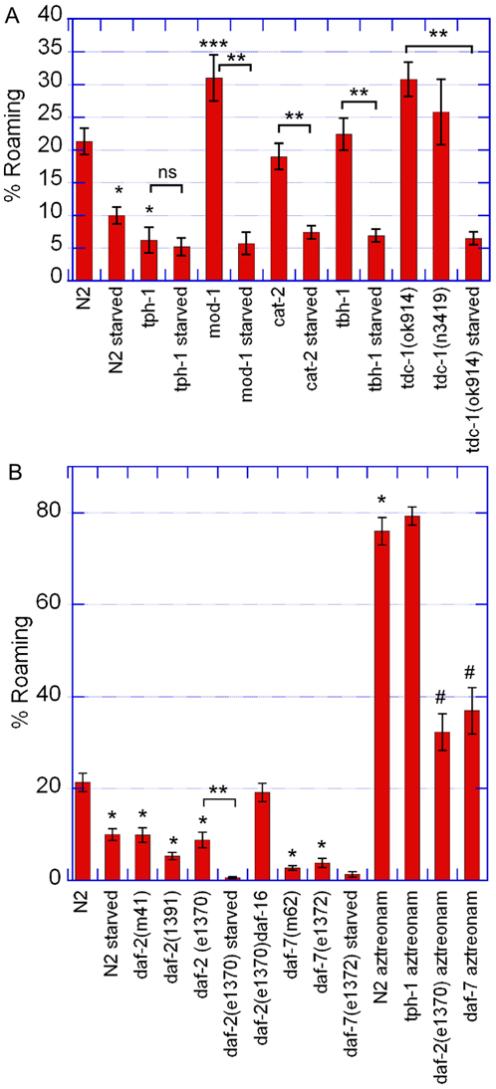
Biogenic amines, insulin and TGF-beta signaling mutants. (A) Percentage of time spent roaming for well-fed and fasted biogenic amines mutants in standard conditions. (B) Percentage of time spent roaming for well-fed and fasted insulin and TGF beta signaling mutants in standard conditions and on aztreonam-treated OP50. At least 20 worms were recorded per condition. Results are presented as mean ± s.e.m. *Different from wild type, Student's t-test p<0.01. ***Different from wild type p<0.05. **Different from each other p<0.01. ^#^Different from wild type on atreonam treated bacteria p<0.01.

Different biogenic amines mutants had very different phenotypes (Fig. 4). Dopamine deficient *cat-2*, octopamine deficient *tbh-1* and tyramine deficient *tdc-1* mutants did not have significant defects. On the contrary, serotonin deficient *tph-1* mutants were strongly defective in roaming.

As serotonin and dopamine are involved in enhanced and basic slowing behavior on food [11], we also analyzed the behavior of fasted biogenic amines mutants. Fasted *cat-2, tbh-1* and *tdc-1* mutants exhibited a wild type starvation response and decreased strongly the time they spent roaming when they reencountered food (Fig. 4). On the contrary, well-fed and fasted *tph-1* mutants did not show significantly different behaviors. Serotonin deficient worms may then behave as if they were starved in all conditions.

To define if the serotonin deficient mutants were still able to assess correctly the presence of nutritional food we analyzed the behavior of *tph-1* mutants on aztreonam treated bacteria. *tph*-1 mutants displayed a wild type phenotype on non-eatable food, although they had a lower average speed in the active phase (Fig. 4). This result shows that these mutants are able to move and that their defect is restricted to high quality food and is not linked to a basic locomotory defect.

MOD-1 is a serotonin receptor involved in the enhanced slowing response [17]. We hypothesized that this chloride channel could also mediate serotonin effect on roaming behavior. We measured roaming time proportion of well-fed and fasted *mod-1* mutants. We observed that well-fed *mod-1* mutants roamed more than *tph-1* mutants and even slightly more than wild type worms. This effect is weak but significant. Thus, serotonin could have antagonistic effects on this locomotory behavior through different receptors. Moreover, *mod-1* mutant animals do not show a defective response to starvation (Fig. 4). Therefore, MOD-1 does not mediate the enhanced dwelling after starvation that is controlled by serotonin and this behavior may then be different from the “enhanced slowing response” [11].

### Insulin and TGF-beta signaling are necessary to induce roaming

Upon starvation, *C. elegans* larvae undergo developmental arrest at an alternative resistant dauer larval stage. Insulin and TGF-beta signaling pathways are involved in dauer formation, quiescence behavior and metabolism regulation and are then related to food signaling [18], [19]. Mutants of the insulin receptor *daf-2* and of the TGF-beta peptide gene *daf-7* were deficient roamers in standard conditions. The *daf-2(e1391)* allele showed a stronger defect than the *daf-2(e1370)* and the *daf-2(m41)* alleles as expected from previous studies focusing on different behaviors [20]. *daf-2* mutants responded to a fasting experience like wild type animals by decreasing even more the time they spent in the active phase (Fig. 4).

More interestingly, *daf-7* and *daf-2* mutants behavior on aztreonam-treated bacteria was altered compared to wild type (Fig. 4). These mutants roamed much more on non-eatable bacteria than on regular food but still dwelled most of their time even in unfavorable food conditions. Therefore, even though these mutants are able to respond to the presence of nutritional food, they are defective in roaming in both food conditions. Some *daf-2* mutants have been shown to have temperature sensitive reduced motility [20]. Here in the roaming phase *daf-2* mutants had a normal sinusoidal movement and speed at 20°C and the defect was due to enhanced time spent in the dwelling phase and not uncoordinated movement (Speed (N2, aztreonam) = 0.158±0.008 mm/s, Speed(*daf2(e1370)*, aztreonam) = 0.148±0.004 mm/s, the difference is not significant, Student's t-test p = 0.3).

In favorable food conditions different insulin peptides activate the DAF-2 insulin receptor. DAF-2 acts through a signaling pathway to inactivate the FoxO transcription factor DAF-16 and prevent dauer entry [18]. In order to check if the roaming behavior was also regulated through the DAF-2/DAF-16 pathway we recorded the trajectories of *daf-2 daf-16* double mutants. The daf-16 mutation was enough to rescue the daf-2 defect and the double mutants showed a wild-type phenotype for the roaming/dwelling behavior (Fig. 4).

Insulin signaling through the DAF-2/DAF-16 pathway and TGF-beta signaling are then required for the animal to balance its time appropriately between dwelling and roaming for all food conditions.

## Discussion

Here we describe new characteristics of *C. elegans* locomotory behavior on its standard laboratory food *Escherichia coli*. We used a new quantitative method of analysis using two parameters of the worm trajectory. As in previous studies we could show that *C. elegans* exhibits two activity phases on food [6]. We show that this behavior depends on the concentration of food and previous food experience. We have established that internal sensing of food presence is necessary to induce dwelling and that chemosensory perception promotes roaming. We have also shown that serotonin signaling and the insulin and TGF-beta pathways control this behavior.

We show that *C. elegans* can vary the time allocated to each phase depending on the food concentration available and that the two-state behavior does not occur in absence of food. Comparably, foraging strategies of *Drosophila melanogaster* have also been shown to vary depending on the food quantity present in the environment [21]. We have also shown that internal perception of food presence is necessary to induce the dwelling state. On the contrary, external sensory perception is not sufficient to induce dwelling on food and even promotes roaming. More precisely, the olfactory neuron AWC seems to promote roaming on food.

These results and the fact that worms modulate the proportion of time spent roaming depending on food nutritional quality [7] show that this behavior is strongly food-related. However, other sensory modalities could be integrated in the regulation of this behavior. It has been shown that some *C. elegans* natural strains that display social behavior are sensitive to high concentration of oxygen. They are hyperactive at 20% of oxygen on food compared to non-social strains but do not behave differently off food [22], [23]. For these social strains oxygen perception could dramatically switch the dwelling/roaming balance. High temperatures or repulsive odors could have the same consequences. Thus, the dwelling and roaming balance must be a subtle and very integrated foraging strategy that is environmentally controlled.

We also show that this behavior depends on previous food experiences of the worm. If the worm has experienced bad conditions before finding a favorable environment it will reduce its probability to roam, maybe in order to stay longer in the best area. This reduced roaming lasts for at least one hour after refeeding. The worm foraging strategy may then be a memory dependent behavior that relies on both its current environment and the environments it has experienced previously.

We have also shown that the time allocation between dwelling and roaming is regulated through the insulin DAF-2/DAF-16 pathway and the TGF-beta DAF-7 pathway. These two pathways are key regulators of dauer transition and of quiescence behavior on high-quality food after fasting [8], [18]. Quiescence and dwelling are different behaviors and we did not see any quiescence events in our experiments on OP50 bacteria. On high quality food, wild type worms actually exhibit a first phase of dwelling and then become quiescent [8]. Then, as insulin and TGF-beta signaling control both behaviors, they could be different responses to different levels of satiety.

Besides, insulin and TGF-beta signaling pathways do not mediate response to short term fasting and are necessary to observe a wild type dwelling behavior in favorable and unfavorable environments. On aztreonam-treated bacteria, wild type worms seem to perceive a very low concentration of eatable bacteria that could correspond to a very low percentage of bacteria that are small enough to be eaten. Insulin and TGF-beta deficient worms are then hypersensitive to food perception even at very low food concentration. As the two pathways act in part in the amphid neurons, TGF-beta and insulin deficient mutants could then have reduced sensory signaling promoting roaming which would change the dwelling/roaming balance to increase the dwelling time proportion.

We have also shown that serotonin mutants are very defective in their time allocation. However, serotonin is not required for food perception as *tph-1* mutants exhibit a wild type phenotype on non-eatable food. We also show that serotonin regulates the modulation of behavior after a short starvation experience, as could be expected from results on the enhanced slowing response behavior [11]. However, the mod-1 serotonin receptor that has been shown to mediate the enhanced slowing response does not promote the one-hour dwelling starvation response (Fig. 4). Therefore, these two changes of behavior after starvation do not seem to be mediated by the same serotonin pathways [17]. Unexpectedly, well-fed mutants of the serotonin receptor *mod-1* showed a weak phenotype opposite to serotonin biosynthesis *tph-1* mutants on regular food. Serotonin signaling controlling this behavior may be mediated by different receptors and serotonin could have antagonistic effects on this behavior. Therefore it would be interesting to understand which receptors mediate serotonin signaling for this behavior in order to gain insight into the neuronal circuits involved. Similarly, serotonin has been shown recently to have antagonistic effects on *C. elegans* egg-laying behavior through different receptors [24].

Besides, *tph-1* mutants have reduced insulin and TGF-beta signaling [16]. One of the effects of serotonin on the dwelling and roaming behavior could then be linked to regulation of the DAF-2 and DAF-7 pathways. However, serotonin role cannot be reduced to this effect, since *tph-1* mutants do not respond to a short starvation period whereas *daf-2* and *daf-7* mutants do. Insulin and TGF-beta signaling pathways are transcriptional pathways. Thus, they must mediate slow metabolic changes, whereas serotonin and other biogenic amines signaling can occur fast and allow the worm to adapt its behavior to faster changes in the environment. Insulin and TGF-beta signaling may then mediate response to long-term environmental changes, such as long starvation periods, whereas serotonin could also control responses to short fasting intervals.

**Figure 5:**
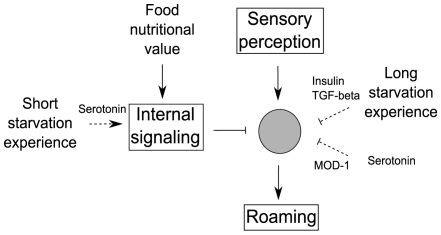
Proposed schematic representation of the control of the dwelling and roaming behavior by sensory perception and internal signaling.

Finally we propose the following representation of our findings (Fig. 5). Food nutritional value is assessed through internal metabolic signaling. The dwelling/roaming time allocation then results from the integration of sensory signals promoting roaming and internal food signals suppressing roaming. Short-term starvation memory mediated by serotonin would enhance suppression of roaming when reentering food. TGF-beta and insulin signaling could define the metabolic state of the worm and allow the animal to adapt to slow changing feeding states. The animal would then get more responsive to food as levels of insulin and TGF–beta signaling gets lower. It would now be interesting to define in which cells these different signaling pathways are integrated and to characterize more precisely the role of serotonin by studying the receptors and the cellular circuits involved.

## Materials and Methods

### *C. elegans* maintenance

Nematodes were cultured on OP50 bacterial lawns on nematode growth medium (NGM) plates at 20°C. N2 Bristol was used as the wild type reference. The following strains were analyzed: *cat-2(e1112)II, ceh-36(ks86)X, ceh-36(ky640)X, che-1(p679)I, che-1(p692)I, che-2(e1033)X, daf-2(e1370)III, daf-2(m41)III, daf-2(e1391)III, daf-2(e1370)III daf-16(mgDf50)I, daf-7(e1372)III, daf-7(m62)III, mod-1(ok103)V, tbh-1(ok1196)X, tdc-1(n3419)II, tdc-1(ok914)II, tph-1(mg280)II, ttx-1(p767)V, ttx-3(ot22)X, ttx-3(ks5)X*.

### Video Analysis

On average 7 worms were transferred to a 60 mm NGM plate spread with bacteria and the whole plate was recorded for up to 2 hours on a custom set-up consisting of a camera macro-objective and a 700x1300 pixels Hamamatsu camera controlled via Labview. For each experiment we recorded 20000 images taken every 400 ms. We used no more than 10 worms per plate to prevent too many possible interactions between them and to track them easily for a long time. The plate was illuminated homogeneously from below with a multi-LED device and the whole set-up was included in a temperature-controlled enclosure. All experiments were performed at 20°C. Worms trajectories were extracted from the recordings via a custom-written code in Matlab. We applied an adaptive threshold method to the first image. The resulting objects in the image were filtered for size and shape in order to find the worms. Each worm-object was then tracked during the 20000 images. Here we recorded the coordinates of the center of mass of each worm in each image during the recording. Each worm trajectory was then automatically truncated into 10 s intervals. The average speed (S) and curvature (C) along the trajectory during each of these intervals were also calculated with Matlab. Speed was defined as the distance between two consecutive points in the trajectory divided by 400 ms. Curvature was defined as the complementary of the angle defined by three consecutive points in the trajectory, which is equivalent to the change in direction.

(S, C) values for all the worms in a given experiment were plotted in the (S, C) plane (Fig. 1E). We repeated each experiment on 5 to 10 different plates. In each condition we recorded the trajectories of at least 20 worms, for a total length of at least 20 hours. We could define two clusters and the (S, C) plane was divided in 2 areas: the roaming zone and the dwelling zone. The clustering boundary (Line A (Fig. 1E)) was defined for wild type worms in standard conditions and was then used for all genotypes and conditions. The roaming percentage for a given experiment was calculated as the number of points in the roaming cluster divided by the total number of points. Data for a given genotype are presented as the mean of the results of the different experiments and the error bars represent the standard error of the mean. Significance was determined using Student's two tailed *t*-test for comparison of the behavior of one mutant strain with wild type behavior. One-way ANOVA followed by a Bonferoni post-hoc test was used for multiple comparisons, such as for comparison of wild type behavior with two different alleles of the same gene.

For visualization, we used data density maps corresponding through a color map to normalized 2D histograms of all the data for a given genotype using 100 bins in each direction.

### Worm handling

L4 larvae were isolated on seeded NGM plates the day before recording. Except for recordings on controlled concentration of bacteria or with aztreonam treatment, experiments were done on NGM plates on which fresh OP50 had been uniformly spread the day before the experiment. The worms were transferred on the experiment plates 10 minutes before the start of recording to cut off the first minutes during which the worm may have responded to perturbations that could have occurred during transfer. For each condition at least 20 worms' trajectories were recorded. The experiments on controlled concentration of OP50 were performed on NGM-streptomycin plates (antibiotic concentration: 50 µg/ml). The day before the recording, OP50 were grown to saturation then blocked for 40 minutes at 37°C with streptomycin at 50 µg/ml. At this concentration, bacteria cannot grow but are not lysed. The concentration of bacteria was derived from optical density (OD) measurements. OD measurements were calibrated by spreading diluted cultures of known OD on LB plates and counting the number of colonies that had grown on the plate. A controlled number of bacteria was then evenly spread on NGM-streptomycin plates. These plates were then seeded with a controlled concentration of bacteria that did not change in time. Experiments with aztreonam were prepared following Gruninger & al protocol [14]. The day before the experiment, OP50 were grown in LB+aztreonam (10 µg/ml) to saturation, and then spread on NGM-aztreonam (10 µg/ml) plates. The day of the recording, the plates were checked for the presence of long “snakes” of *E. coli*. The normal bacteria were washed off the worms by transferring the worms in M9 and then on the recording plate prior to recording.
